# Beta Diversity of Demersal Fish Assemblages in the North-Eastern Pacific: Interactions of Latitude and Depth

**DOI:** 10.1371/journal.pone.0057918

**Published:** 2013-03-19

**Authors:** Marti J. Anderson, Nick Tolimieri, Russell B. Millar

**Affiliations:** 1 New Zealand Institute for Advanced Study, Massey University, Albany, Auckland, New Zealand; 2 Conservation Biology Division, Northwest Fisheries Science Center, National Oceanic and Atmospheric Administration, Seattle, Washington, United States of America; 3 Department of Statistics, University of Auckland, Auckland, New Zealand; National Institute of Water & Atmospheric Research, New Zealand

## Abstract

Knowledge of broad-scale global patterns in beta diversity (i.e., variation or turnover in identities of species) for marine systems is in its infancy. We analysed the beta diversity of groundfish communities along the North American Pacific coast, from trawl data spanning 32.57°N to 48.52°N and 51 m to 1200 m depth. Analyses were based on both the Jaccard measure and the probabilistic Raup-Crick measure, which accounts for variation in alpha diversity. Overall, beta diversity decreased with depth, and this effect was strongest at lower latitudes. Superimposed on this trend were peaks in beta diversity at around 400–600 m and also around 1000–1200 m, which may indicate high turnover around the edges of the oxygen minimum zone. Beta diversity was also observed to decrease with latitude, but this effect was only observed in shallower waters (<200 m); latitudinal turnover began to disappear at depths >800 m. At shallower depths (<200 m), peaks in latitudinal turnover were observed at ∼43°N, 39°N, 35°N and 31°N, which corresponded well with several classically observed oceanographic boundaries. Turnover with depth was stronger than latitudinal turnover, and is likely to reflect strong environmental filtering over relatively short distances. Patterns in beta diversity, including latitude-by-depth interactions, should be integrated with other biodiversity measures in ecosystem-based management and conservation of groundfish communities.

## Introduction

An essential goal in community ecology is to describe large-scale patterns of biodiversity [1], [2], [3]. Perhaps the most well-known of all such patterns is the latitudinal gradient in alpha diversity – a decrease in species richness is observed with increasing latitude [4], [5], [6]. Less well-studied is beta diversity [7], [8]. Anderson et al. [9], following Vellend [10], outlined two different conceptual types of beta diversity. One is non-directional *variation* in species' identities or community structure among sample units within a given area or region at a given spatial (or temporal) scale [7], [11]. The other is *turnover* in community structure or species' identities along a spatial, temporal or environmental gradient [7], [12]. Global patterns in beta diversity along large-scale gradients, such as altitude, depth, latitude and longitude, are not only not yet well documented, they are also less consistent, varying substantially among different ecosystems and types of assemblages of organisms [13], [14].

Studies of beta diversity can inform management [15], as heterogeneity in communities tends to reflect heterogeneity in habitat [7]. Scientific conservation strategies can use measures of beta diversity to maintain a patchwork of heterogeneous habitats that can host a variety of species and community types, rather than focusing efforts on simply preserving high values of species richness, *per se* [16], [17]. Sites might have high conservation value not because of the absolute number of species they contain, but because of the variety of different types of niches present, which tends to be strongly reflected by measures of beta diversity [18].

Areas with high variation in communities (hence high beta diversity) can also indicate important spatial or temporal biogeographic transitions [19], [20], complex mosaics of patchy habitats and edge effects [21], [22] or step functions in whole groups of species' responses or tolerances to variations in environmental parameters or disturbances [23]. Characterising species' turnover along existing environmental gradients and identifying fundamental spatial or temporal transitions will be essential if biodiversity is to be adequately conserved across multiple biomes for a wide range of species [24]. Analyses of beta diversity are essential within the call for ecosystem-based fisheries management [25], [26]. For example, understanding beta diversity can help set boundaries for spatial management, where knowledge of transition zones for fauna is critical. In addition, management must face potential future changes in ocean conditions with climate change [27], [28], which can result in shifting species' ranges or increased assemblage heterogeneity, detectable by changes in beta diversity. Areas of rapid turnover can also indicate important boundaries of energy exchange across ecotones, especially ultimately to predict how whole communities might respond to climate change at large scales [29], [30].

In marine environments, studies of latitudinal gradients in alpha diversity (richness) abound [31], [32], [33], and although there is considerable variation among different types of organisms and in different regions in the strength and slope of observed relationships, meta-analyses [34], [35] clearly show an overall pattern of a decrease in richness with increasing latitude, matching what has been observed in terrestrial systems. Evidence from terrestrial systems also suggests a general pattern of decreasing beta diversity with increasing latitude (e.g., [36], [37]), however the overall relationship, when examined across multiple studies and organisms, is generally quite weak, especially in marine systems [14], [38].

Rather than being positively correlated with average temperature, light or productivity, beta diversity appears to be positively linked to areas of transition, where there is not necessarily a high mean value in environmental parameters nor in alpha richness, but rather in the variation of such parameters [13], [39], [40]. Thus, areas of high beta diversity tend to occur where there are steep environmental gradients, patchy habitat transitions per unit area (environmental heterogeneity, e.g., [39]), or where there are strong historical limits to dispersal through physical, biogeographical, or environmental barriers, where end-points of species ranges tend to accumulate [41]. For example, McKnight et al. [13] found high beta diversity in amphibians, mammals and birds at a wide range of latitudes, stretching from north to south, all along the Pacific edge of the continents of North and South America. These areas have high variability in elevation, and contrast with areas of low beta diversity in the more environmentally homogeneous areas of north-eastern South America and the boreal regions of North America. Stegen et al. [40] found a similar result, with high importance of variance in elevation on temporal and spatial patterns of beta diversity for birds in North America.

In marine environments, our current understanding of patterns in beta diversity along the large-scale spatio-geographical gradients of latitude, longitude or depth is in its infancy. There has been no prior systematic joint investigation of patterns in beta diversity with latitude and depth in the ocean, nor any explicit quantification of the comparative strength or rates of species turnover with depth versus latitude. Previous studies have indicated that marine beta diversity (i) decreases with increasing latitude (e.g., [42]), which may be caused by regional (gamma) richness decreasing more rapidly than local (alpha) richness (e.g., [37]); and (ii) decreases with depth [43], [44], [45], as environments become colder and more uniform.

We predicted that depth and latitude would interact in their effects on beta diversity. For example, latitudinal changes in the structure of fish assemblages along the north-eastern Pacific coast depended on depth; more particularly, communities from disparate latitudes were more similar to one another at depth than they were in shallower waters [46]. This is likely caused by assemblages nearer the surface being more highly influenced by environmental parameters that change directly with latitude, such as surface temperature, oceanic currents, sunlight and productivity. Latitudinal turnover is likely to be less strong in deep systems.

Here, we quantified the beta diversity [7], [8] of groundfish assemblages systematically and jointly along the two gradients of depth and latitude across a large region of the north-eastern Pacific. Beta diversity was measured in two ways: as variation and as turnover (*sensu* Anderson et al. [9]), using data obtained from research trawls done by the US National Marine Fisheries Service (NMFS). Our interest lies in both types of beta diversity. We measured variability in species' identities within particular zones of depth and latitude and characterised changes in this variation across the region as a whole. We also modelled the turnover in community structure of fishes along the depth gradient and along the latitudinal gradient for this coastline.

In terms of *variation*, we predicted that heterogeneity in species' identities would decrease with depth [44], where the environment becomes more homogeneous (dark, cold and highly pressurized). Changes in assemblage variability with depth should be more marked at lower latitudes, where average environmental surface conditions (such as temperature) differ more strongly from deeper depths than they do at higher latitudes.

With respect to *turnover*, we predicted an interaction, namely that: (i) the turnover in the structure of communities along the depth gradient would be stronger at lower latitudes than at higher latitudes; and (ii) latitudinal turnover would be stronger at shallower depths than at deeper depths. We used a recently developed form of distance-decay models [47] to test these predictions.

It is known that patterns in beta diversity can be affected by changes in the number of species or alpha diversity [48], [49], [50]. The presence of sparser assemblages will tend to reflect greater dissimilarities due to differences in alpha diversity alone [51]. For example, an assemblage containing 2 species and an assemblage containing 10 species can share no more than 20% of the total number of species present in both assemblages. Variation in alpha diversity with depth and latitude has already been documented for these assemblages [52], and we wished to ensure that beta diversity analyses were independent of alpha variation. Thus, we analysed beta diversity as heterogeneity using not only the classical Jaccard resemblance measure, but also using the probabilistic Raup-Crick measure [53], in order to control for differences in alpha diversity expected to occur within different depth and latitude zones.

## Materials and Methods

### Groundfish trawl data

**Figure 1:**
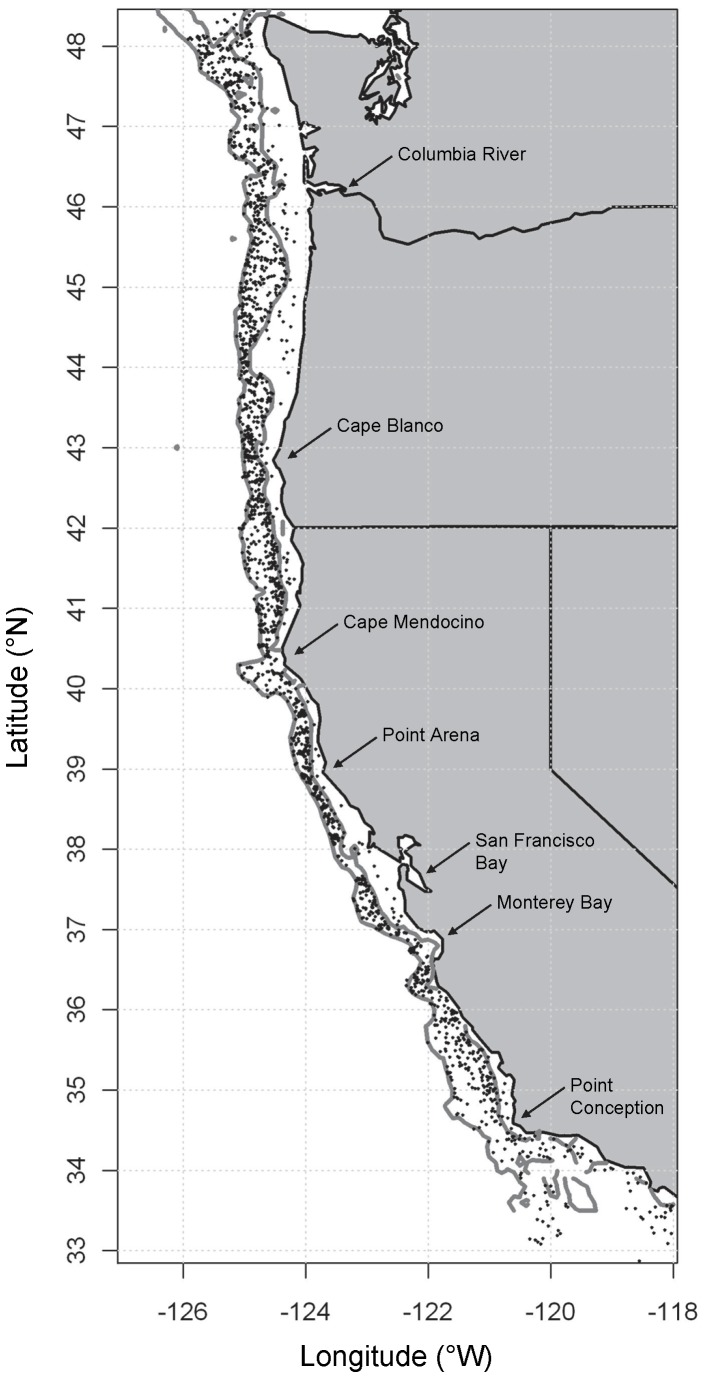
Map showing positions of trawls. Map of the west coast of the United States, showing positions of 1,974 sites from trawl datasets (small black dots) that were included in this study, along with bathymetry lines (in grey) at 200 m and 1200 m depth.

Data were obtained from the annual research bottom-trawl surveys done by the Northwest Fisheries Science Centre, NMFS over the period from 1999–2003, including the Pacific West Coast Upper Continental Slope Trawl Survey [54] and the U.S. West Coast Bottom Trawl Survey [55]. Further information regarding these databases, held by NOAA, can be obtained by contacting Beth Horness (beth.horness@noaa.gov). Previous work has demonstrated that inter-annual variability in these assemblages, at least over this period, was unimportant relative to the strong effects of temperature, depth, latitude and longitude [46]. Thus, as in previous analyses of biodiversity for these assemblages [46], [52], [56], we did not explicitly account for temporal variation in what follows. We restricted our analyses here to trawls >50 m in depth, yielding a total of 1,974 sample trawls over the period from 1999–2003, and these ranged from 32.57°N to 48.52°N in latitude and from 51 m to 1341 m in depth (Fig. 1). The duration of trawls was ca. 15 min at a speed of 2.2 knots, towing Aberdeen style nets with a small mesh liner (≤5 cm) in the cod-end. Only fish identified to species level (as per [57]) were included in analyses. Bottom trawling targets demersal fishes, but some pelagic fishes are also caught in nets during lowering and retrieving. Preliminary investigations indicated that omission of pelagic fishes had no appreciable effects on results, so pelagic fishes were not removed from the database. As complex rocky habitats are not surveyed by trawling, inferences are necessarily limited to fishes obtained from trawlable habitats across the region sampled.

### Beta diversity as variation

To measure variation in species' identities among sample units within particular combinations of depth and latitude, we binned trawls into 200 m-depth×2°-latitude cells across the study region (Table 1). The majority of the cells had >20 hauls (sample units), which we considered more than sufficient to measure beta diversity as variation. However, along the margins of the regions' sampling extent, there were fewer samples. We did not analyse variation for cells having fewer than 10 sample units; thus, trawls at depths greater than 1200 m or at latitudes above 48°N were excluded (Table 1). There were 10–20 sample units available, however, for cells within the lowest latitudinal band (i.e., 32°N–33.99°N). These cells were therefore included in analyses, but should be interpreted with some caution. Note that the measures of variation used for this study (see below) are like the classical univariate unbiased estimators of either variance or standard deviation. They become more precise with increasing sample size, but are not biased in any way by reference to the number of samples. Thus, differences in the number of trawls within different cells across the region do not pose a problem for comparative analyses of beta diversity as variation.

**Table 1:** Sample sizes (number of trawls) in the 2° latitude×200 m depth cells for analyses of beta diversity as variation.

	Latitude (°N)								
Depth (m)	32	34	36	38	40	42	44	46	48
50–200	19	32	21	25	28	26	29	44	30
200–400	18	60	55	68	58	73	77	48	13
400–600	17	56	35	63	52	63	67	52	6
600–800	13	30	36	41	47	44	43	43	1
800–1000	15	24	22	23	38	28	29	42	3
1000–1200	11	24	38	32	39	38	43	38	1
1200+	2	2	5	3	8	16	5	12	0

Variation in the identities of fish species within each cell was measured as the average Jaccard dissimilarity calculated among all pairs of trawls. Thus, within a cell having *n* sample points, this measure is:



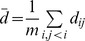
, where *d_ij_* indicates the dissimilarity between the *i* ^th^ and *j* ^th^ pair of sample units (for *i* = 1,..., *n* and *j* = 1,..., *n*) and *m* = *n*(*n*–1)/2, which is just the number of dissimilarity pairs. Analyses were also done using the square root of a pseudo multivariate component of variation (as in [58], [59]); that is,



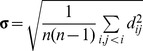
.

However, this measure yielded results having a correlation of 0.999 with those obtained using the average, so we therefore only show results obtained using the average dissimilarity, for ease in interpretability. The Jaccard measure is directly interpretable as the proportion of unshared species between two sample units; it is also sometimes expressed as a percentage, by multiplying ×100.

The Jaccard dissimilarity is known to be affected by differences in the relative richness of samples. For example, if one trawl has 4 species and another has 10 species, the greatest possible overlap in shared species is 4/10, which limits the potential Jaccard similarity to an upper bound of 0.40; equivalently the Jaccard dissimilarity will have a lower bound of 0.6. To control for the effect of differential alpha diversity on measures of beta diversity, we also calculated the Raup-Crick dissimilarity measure [53] among all pairs of sample units. This is a probabilistic coefficient that examines the calculated Jaccard value of *d_ij_* for any particular pair by comparison to the distribution of possible values that it could take if the number of species present in the two sample units (α_1_ and α_2_, say) were drawn at random from the list of species present in the larger species pool (gamma diversity, γ). For our purposes, the species-pool was defined as the list of species present in the depth-by-latitude cell within which the two sample units occurred. The number of species in each list was therefore treated as the gamma diversity for each cell in the study.

Measures of beta diversity as variation can also be strongly affected by difference in the area of the sampling extent – a consequence of the well-known species-area relationship [60], [61]. We expect the number of species (alpha diversity) to increase and also the likelihood of encountering a greater variety of communities (hence beta diversity) to increase with increasing area sampled [62]. The depth-by-latitude cells were constructed expressly on the basis of equally spaced depth and latitude zones, but the areal extent of each cell depended on the bathymetric contours of the shelf and slope along the coast. In addition, the spatial extent of the trawls done within a cell may not necessarily completely cover the areal extent of the cells, as defined.

To obtain the sampled areal extent within each cell, we calculated the convex hull of the spatial distribution of the trawls, using the latitude and longitude values and great-circle distances from a simple round-earth model. Latitude was first transformed to kilometres, using the constant conversion factor of 111.325 km per degree. Distances for longitudes vary with latitude. The conversion factor used for longitude (in km) was therefore calculated separately for each cell as: *long* = cos(*lat*) × 111.325, where *lat* was the median value of latitude, expressed in radians, from the set of coordinates for the trawls within that cell. After conversion, the area sampled within each cell was calculated as the area of the convex hull defined by the set of coordinates for the trawls within that cell.

A second method, relying on a variety of available bathymetric data layers in GIS, was also used to calculate the areas sampled within each depth-by-latitude cell. Bathymetric data sources included the U.S. National Ocean Service Hydrographic Database, the U.S. Geological Survey (USGS), the Monterey Bay Aquarium Research Institute, the U.S. Army Corps of Engineers, and various other academic institutions. Topographic data were also obtained from the USGS and the Shuttle Radar Topography Mission (SRTM). The results and interpretations obtained using areas measured in this way did not differ substantially from the results obtained using the method described above. Thus, for simplicity, these additional analyses are not included here.

The average number of species per trawl (alpha diversity,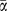), the total number of species per cell (gamma diversity, γ) and average Jaccard or Raup-Crick dissimilarity (beta diversity, 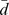) were each examined for their potential relationship with area sampled across all cells. Approximately linear relationships were found between beta diversity measures and area (see Results). Patterns in beta diversity with latitude and depth were therefore also examined after controlling for variation in the area sampled, using residuals from these linear models.

### Beta diversity as turnover

The degree of turnover in species' identities along a spatial, temporal or environmental gradient can be measured as the slope from a distance-decay model [9], [12], [36], [47]. We measured turnover along the depth gradient by first dividing the data up into a series of 1° latitudinal bands, then plotting the pair-wise Jaccard similarities (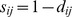) between sample units against the pair-wise absolute differences in depth, separately within each latitudinal band. For each of these, we then fit a distance-decay model to the points as a binomial log-linear generalised linear model (GLM), as described in Millar et al. [47]. Namely, E[*s*] = *τe* ^−*βx*^, where *x* is the absolute difference in depth. After taking logs, this gives: log(E[s]) = *t*−*βx*. The intercept (*τ* = exp(*t*)) from such a model is interpretable as the similarity of two sample units within the same depth, which we shall refer to here as a “nugget”, following the language of semi-variograms in geo-statistics. This method is advantageous in that it models the similarities directly, requiring neither any correction for nor omission of zero values of similarity (see [47] for details).

As the inter-sample similarities are not independent of one another, standard errors for the estimated slope (*β*) and intercept (*τ*) for each model were obtained using a leave-one-out jackknife procedure on the original sample units [47]. The statistical significance of the relationship was tested in each case using a Mantel test on the basis of the Spearman rank correlation (rho, *ρ*) between *s* and *x*, using 9999 permutations of the original sample units (trawls) within each latitudinal band. We also calculated the halving distance, defined as the distance along the gradient that would yield a halving in the similarity [7]. Specifically, halving distance is *h* = −log(0.5)/*β* = 0.693/*β* [47]. The values for the slope (turnover with depth), intercept (nugget), halving distance and Mantel correlation were then compared across the latitudes.

A similar procedure was done to examine turnover along the latitudinal gradient at different depths. For this, the data were first separated into a series of 100 m depth strata (i.e., 50–150 m, 150–250 m, 250–350 m, etc.). Turnover with latitude was then estimated separately within each depth stratum using distance-decay plots and binomial log-linear GLMs as described above, but where *x* = absolute difference in latitude. The degree of latitudinal turnover was then compared across depths.

All analyses were done using R [63]. The R code for calculating Raup-Crick probabilistic resemblances is given by Chase et al. [51]. The R code for modelling distance-decay relationships, including jack-knife estimation of standard errors, is available in Millar et al. [47].

## Results

**Figure 2:**
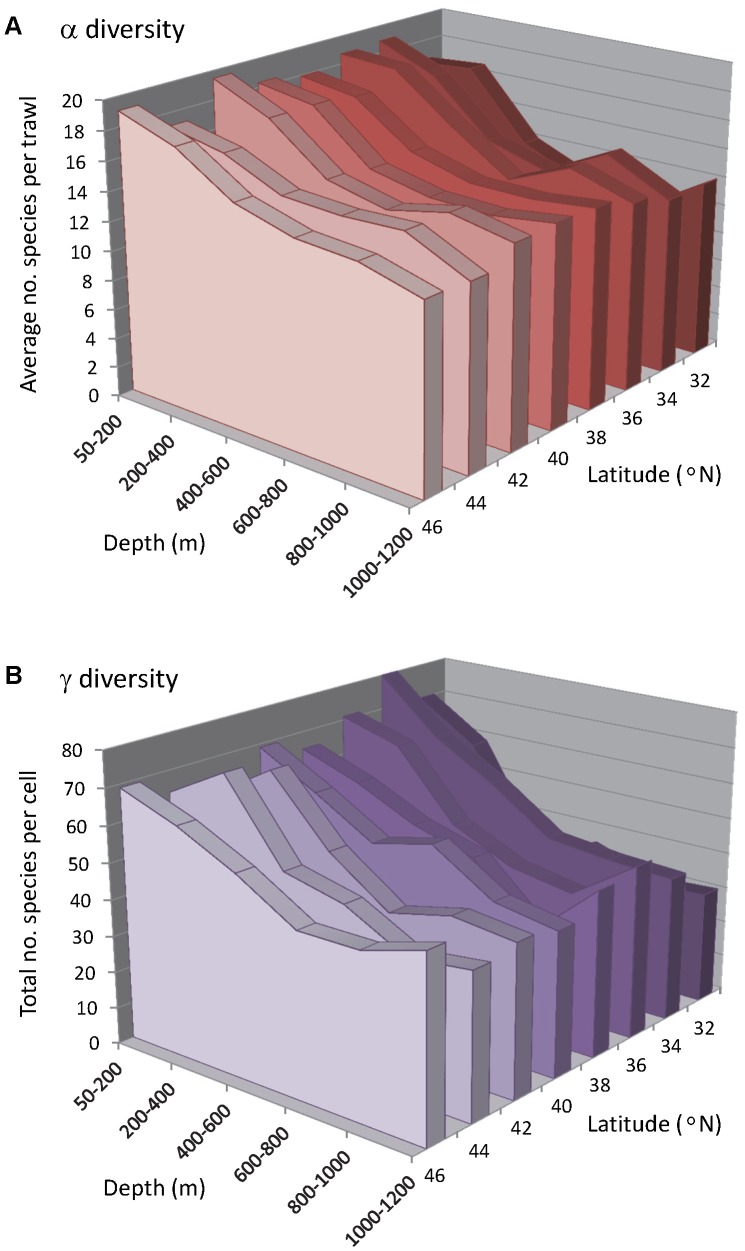
Alpha and gamma diversity *versus* depth and latitude. Trends with depth and latitude in (**A**) the average number of species per trawl (alpha diversity) and (**B**) the total number of species (gamma diversity). Values are plotted for trawls within cells consisting of bins made from 200 m depth intervals and 2° of latitude. The standard errors per cell (which, for clarity, are not shown graphically) ranged from 0.2&o 1.25 for (**A**). No standard errors are available for (**B**), as there is only one value of γ obtained per cell.

There were 243 fish species recorded from the 1,974 trawls in this dataset. The average number of species per trawl (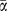) and the total number of species (γ) per depth-by-latitude cell varied across the studied region (Fig. 2a). At high latitudes (e.g., 46–47.99°N), 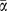 tended to decrease gradually with depth, from an average of 19.05 (± 0.63 s.e.) species per trawl in the shallow depth zone (<200 m) to 12.58 (± 0.40 s.e.) species in the 1000–1200 m depth zone. At lower latitudes (e.g., from 32–38°N) there was a pattern of an initial rather steep decrease in 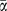, with minima reached around 600–800 m depth, followed by a slight increase at deeper depths of 1000–1200 m (Fig. 2a). For example, within the 32–33.99°N latitudinal band, there were only 10.38 (± 0.73 s.e.) species per trawl, on average, at 600–800 m. Concomitantly, there were no obvious differences in 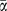 with latitude at shallow or deep depths, but 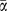 apparently increased with latitude at intermediate depths (Fig. 2a).

The total number of species per cell (γ) was highest in shallow depth zones across all latitudes and generally decreased with depth (Fig. 2b). The average value for γ in the <200 m depth zone was 68.5 species (range = 60–80 species). However, the lowest values for γ tended to occur in the 800–1000 m depth zone (average γ = 38.4, range = 30–45 species), and increased again at the deepest depth zone sampled here of 1000–1200 m (average γ = 42.1, range = 31–50 species). Changes in either γ or 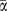 with depth were also clearly more pronounced at the lower latitudes than at the higher latitudes (Fig. 2b).

### Beta diversity as variation

**Figure 3:**
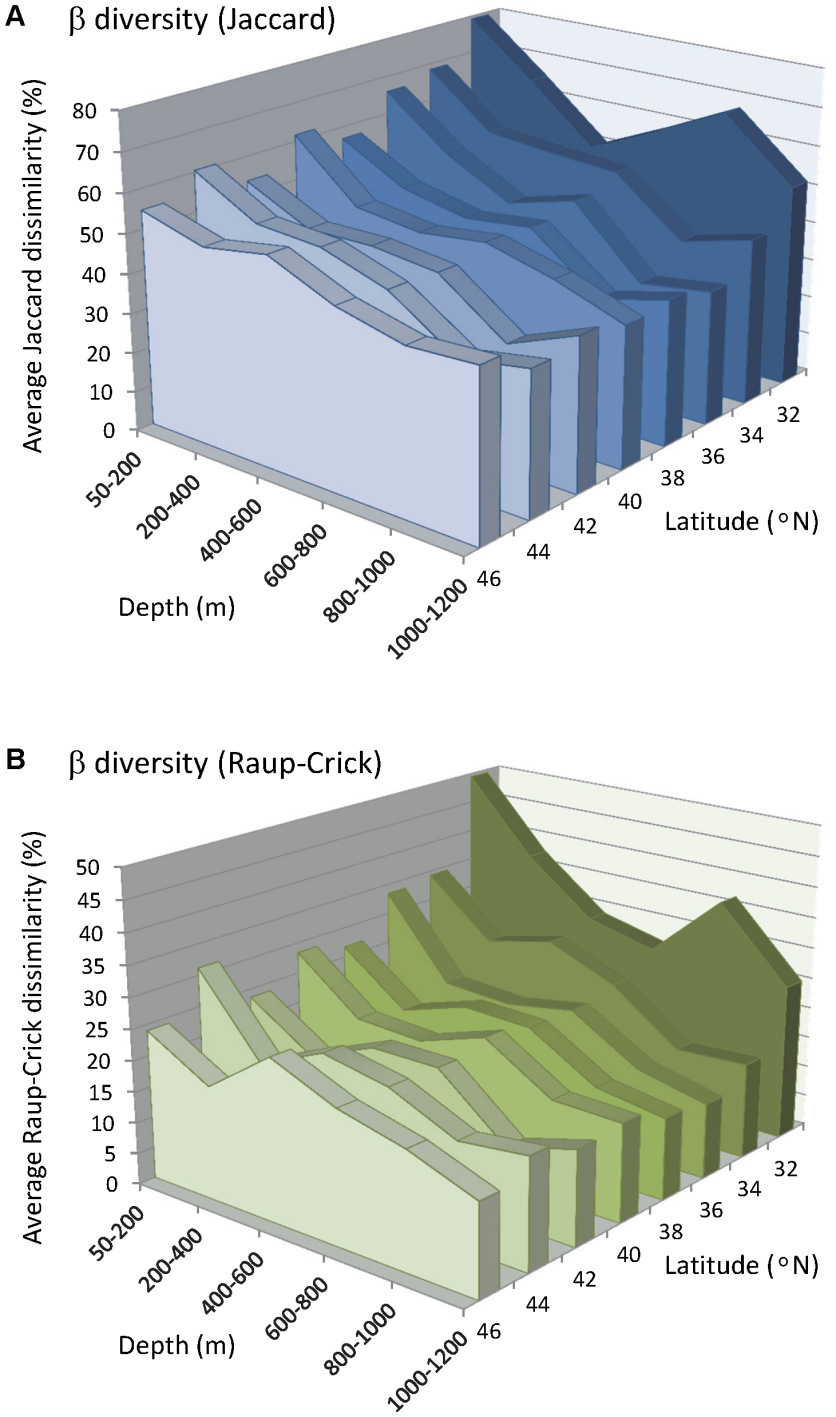
Beta diversity *versus* depth and latitude. Trends in beta diversity with depth and latitude, measured as (**A**) average Jaccard dissimilarity and (**B**) average Raup-Crick dissimilarity, which controls for variation in richness (alpha diversity). Values are plotted for trawls within cells consisting of bins made from 200 m depth intervals and 2° of latitude. The standard errors per cell (which, for clarity, are not shown graphically) ranged from 0.22 to 2.02 for (**A**) and 0.44 to 3.16 for (**B**).

Variation in species' identities, as measured using the Jaccard measure, did tend to decrease overall with depth, but this relationship was by no means linear (Fig. 3a). There was an initial decrease in beta diversity with depth, which then increased slightly to show a “bump” at intermediate depths. Values then decreased again with increasing depth, but an upward trend in variability was then encountered again at the deepest depth zone of 1000–1200 m (Fig. 3a). Interestingly, the intermediate depth at which the “bump” in beta diversity occurred depended on latitude (Fig 3a); it tended to occur around 600 m in mid-latitudes, but was deeper at lower latitudes (e.g., occurring at 800–1000 m for the 32–33.99°N latitudinal band) and shallower at higher latitudes (occurring at 200–400 m for the 46–47.99°N latitudinal band).

Changes in the variation in species' identities with latitude were strongest at the shallowest (<200 m) and deepest (1000–1200 m) depth strata, where beta diversity increased with decreasing latitude. At depths <200 m, the average percentage of shared species among the trawls was only 20.8% (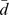 = 79.2%) at low latitudes (32–33.99°N), whereas at a high latitudes (46–47.99°N), the average percentage of shared species was 45.3% (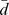 = 54.7%).

Analyses of beta diversity as variation based on the Raup-Crick measure yielded similar results that served to clarify these trends (Fig. 3b). Beta diversity decreased with increasing latitude from 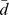 = 49.4% in the south (32–33.99°N) to 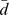 = 23.99% in the north (46–47.99°N) at depths <200 m. The “bump” in beta diversity observed at intermediate depths was also maintained for analyses based on the Raup-Crick measure, occurring at either 400–600 m or 600–800 m for all latitudinal bands (Fig. 3b), except for the lowest (32–33.99°N), where it occurred deeper, at 800–1000 m. The “up-turn” in beta diversity at the bottom of the depth profile sampled here (1000–1200 m) indicated perhaps the beginning of a transition zone which may continue to deeper depths outside of the sampling frame.

### Effects of area

**Figure 4:**
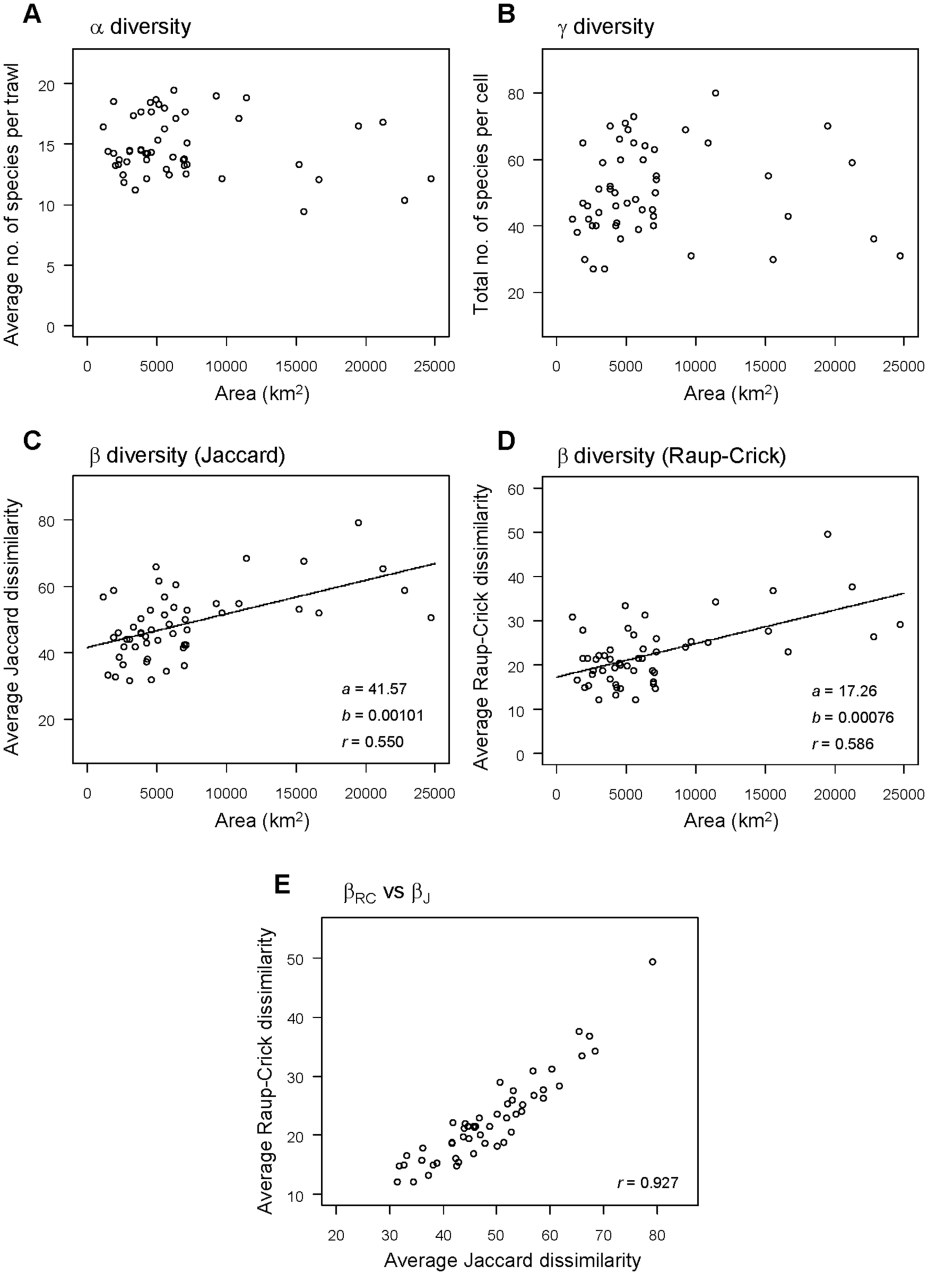
Diversity measures *versus* sampled area. Relationship between area sampled (measured as the convex hull of the coordinates for trawls within a 200 m-depth×2° latitude cell, as great-circle distances) and each of (**A**) average number of species per trawl (alpha diversity); (**B**) total number of species per cell (gamma diversity); (**C**) average Jaccard dissimilarity (beta diversity); (**D**) average Raup-Crick dissimilarity (beta diversity accounting for variation in alpha diversity). Note that the Pearson linear correlation values (*r*) and the intercept and slope coefficients (*a* and *b*, respectively) from a linear regression model are also shown on individual plots, where relevant. Also shown is (**E**) the relationship between beta diversity calculated using Jaccard *vs* Raup-Crick dissimilarity measures.

There was no significant effect of the sampled area within a depth-by-latitude cell on either the average richness per trawl (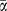, Fig. 4a, *r* = −0.170, *P* = 0.226), or the total number of species obtained from within that cell (γ, Fig. 4b, *r* = −0.027, *P* = 0.848). There was a positive effect of area, however, on beta diversity, whether measured using Jaccard (Fig. 4c, *r* = 0.550, *P*<0.001) or Raup-Crick (Fig. 4d, *r* = 0.586, *P*<0.001). In addition, the values for within-cell variation in species' identities obtained using these two different measures were strongly correlated with one another (*r* = 0.927, *P*<0.001, Fig. 4e), indicating that the effects of variation in alpha diversity on the Jaccard measure were consistent across the study region as a whole.

**Figure 5:**
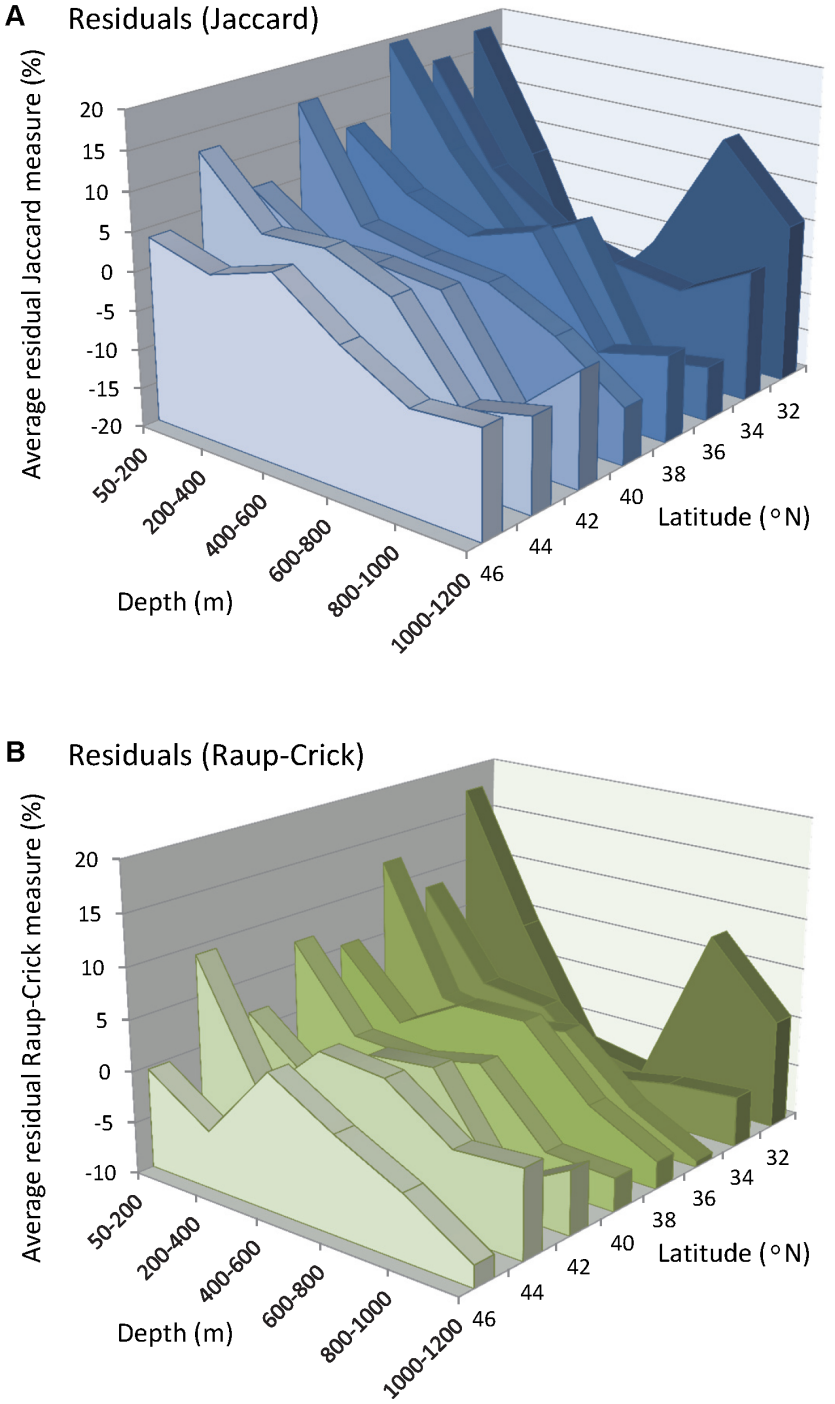
Residual beta diversity, after accounting for area, *versus* depth and latitude. Trends in beta diversity with depth and latitude, after controlling for differences in sampled area, measured as (**A**) the residuals of the average Jaccard dissimilarity; and (**B**) the residuals of the average Raup-Crick dissimilarity, after fitting linear models for each variable *vs* area, as shown in Fig. 4(**C**) and (**D**), respectively. Values are plotted for trawls within cells defined as in Fig. 3.

To control for variation across the study region in the sizes of the areas sampled from each depth-by-latitude cell, residuals were obtained after fitting the linear models of beta diversity calculated using either Jaccard or Raup-Crick *vs* area. The coefficients for these two linear models are given in Figs. 4c and d, respectively. Even after controlling for area, however, the previously observed trends remained, with greater beta diversity found at shallower sites and at lower latitudes (Fig. 5). There was also still a clear secondary peak in beta diversity around 600 m, which was especially pronounced at high latitudes (44–48°N). At low latitudes (32–33.99°N), the drop in beta diversity with increasing depth was the most dramatic, and the secondary peak did not occur until the 800–1000 m depth zone (Fig. 5a, b). In addition, once the variation in alpha diversity was taken into account using Raup-Crick, the increase in beta diversity shown for the deepest depth stratum (1000–1200 m) was either more modest or non-existent (Fig. 5b) compared to the increases seen in the uncorrected Jaccard values at these depths (Fig. 5a).

### Beta diversity as turnover

**Figure 6:**
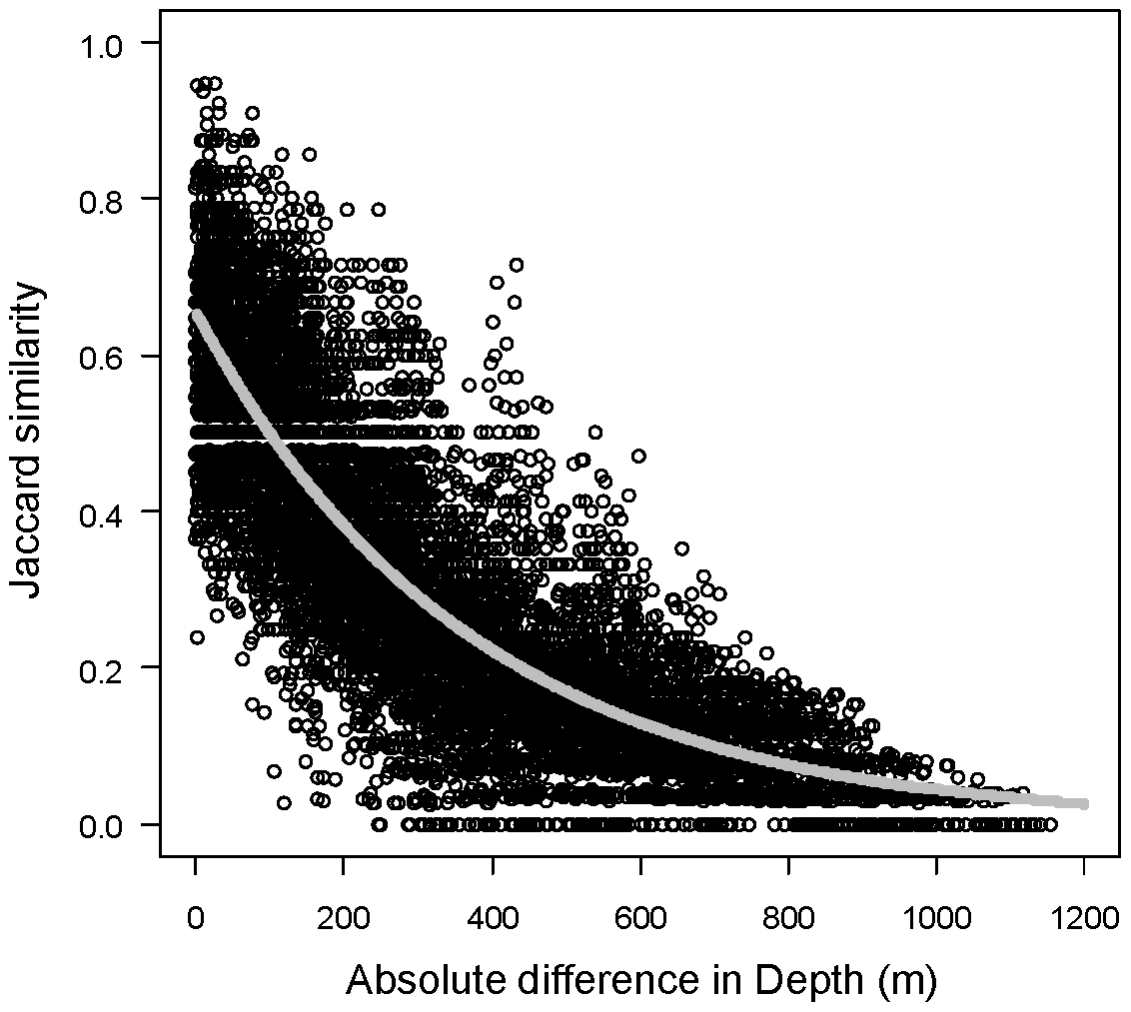
Fish community similarity *versus* differences in depth. Distance-decay plot, showing the decay in similarity of fish communities with increasing absolute differences in depth within the 1° latitudinal band of 35–35.99°N. The fitted model from the binomial GLM with log link is shown in grey. Here, there were 9045 similarity values from 135 trawls. For more details, see the text and Table 2.

A typical distance-decay plot for these data, showing turnover in the identities of fish species with depth, is given for the 1° latitudinal band of 35–35.99°N in Fig. 6. There are a number of trawls having no species in common; these have similarity values of zero and line up along the bottom of the plot. Note also there is a lower bound on the possible non-zero values for the proportion of shared species measured by Jaccard, as well as a clear gap in possible values around 0.5, as species are naturally measured only in integer units. Also shown (in grey) is the fitted distance-decay curve from the binomial log-linear GLM, having estimated slope and intercept of 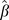 = 0.002698 and 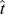 = −0.4229, respectively (Table 2). The value of the curve at a distance (in depth) of zero is the estimated nugget, being 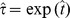 = exp(−0.4229) = 0.655. Thus, it would be expected that the species lists from two trawls taken from the same depth within the latitudinal band of 35–35.99°N would have ∼65.5% of their species in common.

**Table 2:** Results from binomial log-linear GLM fits of beta diversity as turnover along a depth gradient, done separately for each 1° latitudinal band.

Latitude (°N)	No. trawls	No. similarity values	Null deviance	Residual deviance	Residual df	Effective sample size	*r* ^2^	Mantel ρ
32	27	351	80.72	39.19	349	8.90	0.514	0.766
33	68	2278	490.75	271.16	2276	8.39	0.447	0.695
34	93	4278	962.82	475.35	4276	9.00	0.506	0.736
35	135	9045	1937.88	591.52	9043	15.29	0.695	0.855
36	110	5995	1367.71	497.38	5993	12.05	0.636	0.821
37	102	5151	1156.22	508.35	5149	10.13	0.560	0.778
38	123	7503	1477.70	496.41	7501	15.11	0.664	0.847
39	132	8646	1914.79	843.79	8644	10.24	0.559	0.774
40	125	7750	1534.54	642.23	7748	12.06	0.581	0.786
41	146	10585	2378.12	824.74	10583	12.83	0.653	0.833
42	156	12090	2542.88	879.96	12088	13.74	0.654	0.830
43	131	8515	1666.90	700.84	8513	12.15	0.580	0.780
44	145	10440	2391.06	1068.00	10438	9.77	0.553	0.773
45	149	11026	2361.34	939.11	11024	11.74	0.602	0.806
46	134	8911	1920.04	712.29	8909	12.51	0.629	0.816
47	144	10296	2367.99	1073.25	10294	9.59	0.547	0.776
48	54	1431	223.76	82.88	1429	17.24	0.630	0.760

As indicated in Millar et al. [47], the fitting of a binomial GLM model with non-integer response values (in the present case, similarities between 0 and 1), produces a warning message in R, as the routine expects the number of successes to be either 0 or 1 when the number of trials is not specified (and hence is assumed to be 1). In such cases, the residual deviance (*dev*_Res_) divided by the residual degrees of freedom (*df*_Res_) gives a measure of dispersion relative to a Bernoulli trial. For the distance-decay models given here, we have severe under-dispersion, with the residual degrees of freedom being much larger than the residual deviance (e.g., for the data shown in Fig. 6, the value is *dev*_Res_/*df*_Res_ = 591.5/9043 = 0.0654). The interpretation of the inverse of this quantity, *df*_Res_/*dev*_Res_, is the effective sample size, *E_n_*. Thus, for the data shown in Fig. 6, this value is 1/0.0654 = 15 (Table 2). The implication is that the sampling variability of the Jaccard similarity values in this model suggests that there were effectively *E_n_* = 15 trials to produce each data value. Recall that the Jaccard similarity is interpretable as the proportion of shared species. In the binomial GLM context, shared species are a “success”, and the model suggests the effective number of species being trialled to see if they match is 15 for this particular model. This sheds light on the degree of redundancy in information inherent in the multi-collinearity of species' simultaneous responses along the gradient.

Note also that a form of explained variation for these models is the explained deviance, or *r* ^2^ = (1 − *dev*_Res_/*dev*_Null_), where *dev*_Null_ is the null deviance from the GLM. For the data shown in Fig. 6, this is (1 − 591.5/1938) = 0.695 (Table 2). Another useful measure of the strength of the relationship is the Spearman rank correlation ρ (rho) and associated *P*-value for a Mantel test, which was highly significant for the data shown in Fig. 6 (ρ = 0.855, *P* = 0.0001, Table 2).

**Figure 7:**
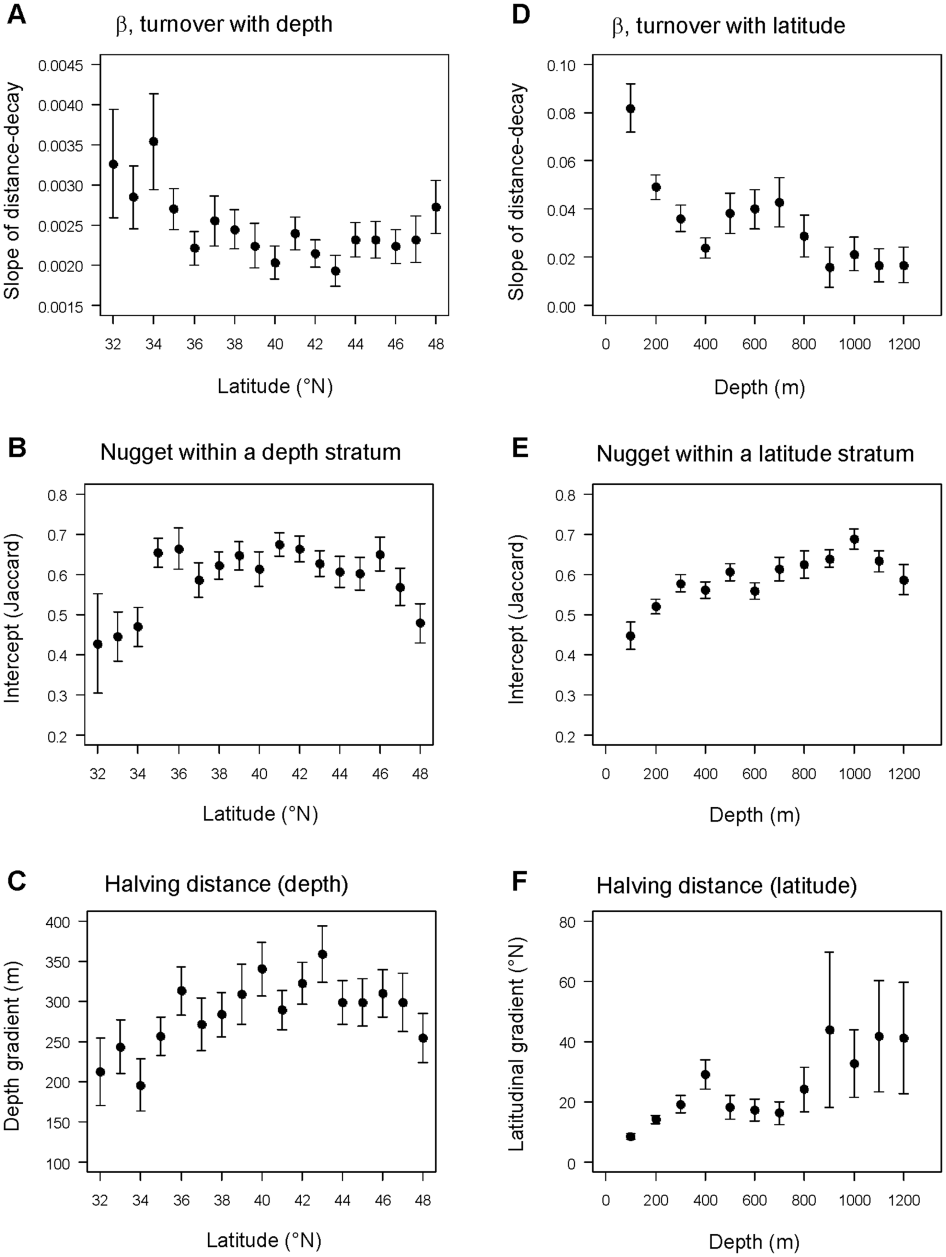
Parameters estimated from distance-decay models. Summary of results for parameters estimated from distance-decay models of turnover in fish assemblages along the depth gradient (**A**, **B**, **C**, calculated separately within each 1° latitudinal band) or along the latitudinal gradient (**D**, **E**, **F**, calculated separately within each 100 m depth stratum). Standard error bars on each of the estimated parameters were obtained using leave-one-out jack-knife resampling. For other details regarding these models, including sample sizes, explained variation and other diagnostics, see Tables 2 and 3.

The degree of turnover in assemblages with depth, as measured by the slope of the distance-decay model, decreased with increasing latitude from 32°N to 36°N (Table 2, Fig. 7a). Turnover with depth levelled off and remained fairly constant between 36°N and 47°N. There was then a slight increase in turnover with depth within the highest latitudinal band (48–48.99°N). The estimated nugget was low (indicating high within-depth-stratum variation) for the lower latitudes, but then increased and remained fairly constant at around 60% similarity between about 35°N and 47°N (Fig. 7b). Halving distance gradually increased with increasing latitude, with peaks (i.e., lowest turnover) being observed for the 40°N and 43°N latitudinal bands (Fig. 7c). Turnover with depth was, overall, very strong and highly significant at all latitudes (*P* = 0.0001), with explained variation ranging from *r* ^2^ = 0.447 to 0.695 and Mantel Spearman rank correlations ranging from ρ = 0.695 to 0.855 (Table 2).

In contrast, the turnover in community structure along the latitudinal gradient was not as strong as it was for depth, with *r* ^2^ ranging from 0.034 to 0.342 and ρ ranging from 0.199 to 0.594 (Table 3). These distance-decay models were all statistically significant, however, by Mantel permutation tests (*P* = 0.0001). Latitudinal turnover was very high at the shallowest depths and decreased rapidly with depth until ∼400 m (Fig. 7d, Table 3). Turnover with latitude then increased within the intermediate depth strata of 500–700 m, before decreasing again at deeper depths (≥800 m). The nugget values, indicating the degree of similarity among communities within a given latitudinal band, gradually increased with depth from 50–1000 m, but dropped slightly for the deepest depth strata (1100–1200 m, Fig. 7e). Estimated values for the nugget parameter within latitudinal bands (Fig. 7e) were similar to those within depth strata (Fig. 7b), with many around ∼60%. Halving distances (Fig. 7f) reflected similar patterns to what was seen for latitudinal turnover using the slope coefficient (Fig. 7d), but there was a marked increase in the variability of the halving distances (estimated using the jack-knife) for latitudinal distance-decay models within the deepest depth strata (≥900 m). These also corresponded to the models having some of the smallest *r* ^2^ values (Table 3).

**Table 3:** Results from binomial log-linear GLM fits of beta diversity as turnover along a latitude gradient, done separately for each 100 m depth stratum.

Depth (m)	No. trawls	No. similarity values	Null deviance	Residual deviance	Residual df	Effective sample size	*r* ^2^	Mantel ρ
100	171	14535	1942.07	1278.03	14533	11.37	0.342	0.594
200	217	23436	1484.18	976.87	23434	23.99	0.342	0.575
300	207	21321	1222.86	924.72	21319	23.05	0.244	0.484
400	263	34453	1949.51	1728.82	34451	19.93	0.113	0.331
500	208	21528	2529.06	2145.53	21526	10.03	0.152	0.412
600	172	14706	1028.22	812.11	14704	18.11	0.210	0.472
700	108	5778	464.07	351.25	5776	16.44	0.243	0.464
800	123	7503	786.18	688.62	7501	10.89	0.124	0.354
900	146	10585	967.65	920.30	10583	11.50	0.049	0.199
1000	124	7626	503.44	439.13	7624	17.36	0.128	0.320
1100	110	5995	358.50	330.34	5993	18.14	0.079	0.251
1200	119	7021	749.96	724.34	7019	9.69	0.034	0.221

## Discussion

### Latitudinal beta diversity

There was a clear interaction in patterns of latitudinal turnover with depth. At shallow depths (<200 m), peaks in beta diversity, hence high turnover, were identified at particular places along the coast: namely, near 43°N (around Cape Blanco), 39°N (around Point Arena), 35°N (around Point Conception) and 33°N (area south of the Channel Islands). Peaks in beta diversity were especially apparent when variation in alpha diversity was specifically taken into account through the use of the Raup-Crick probabilistic measure (Fig. 3b). High variability in species identities around the region of Point Conception was also very clear after the areal extent of the sampling was taken into account (Fig. 5b). These findings correspond very well with the identification of biogeographic boundaries as obtained through analysis of the endpoints of species' ranges, assemblage structure and patterns in alpha diversity [41], [46], [52], [64]. Horn and Allen [41] reported greater numbers of range terminations occurring around 31–33°N, in southern California and northern Baja California, where the range endpoints for species with warm-temperature and cool-temperature affinities overlap [41]. Phylogenetic and range-endpoint analyses suggest that the submerged canyons in the Los Angeles region may act as a barrier for many taxa, while Point Conception is better considered as a transition zone [64], [65], [66], [67], [68]. This is consistent with the high beta diversity found for that area in this study. In addition, the more northerly areas of high beta diversity found in the present study along the Pacific Coast in shallower waters (around 43°N and 39°N) may well be driven by areas of high oceanographic heterogeneity, produced by variation in nutrient productivity, freshwater discharge and/or upwelling [27].

Latitudinal turnover decreased with increasing depth (Fig. 7d), and the latitudes showing peaks in beta diversity in shallow coastal waters were no longer apparent at depth (>200 m). The sharpest decrease in the distance-decay curve was observed between 200 and 400 m, with more modest decreases in beta diversity observed beyond 600 m depth. By around 900 m, the Mantel correlation for latitudinal distance decay dropped to ρ<0.20 (Table 3). This decrease in biogeographic spatial effects with increasing depth was also observed by Price et al. [43], who measured gamma and beta diversity for Asteroids (sea stars) across the whole of the Atlantic Ocean. They observed that, as depth increased, so did the faunal similarity among regions. This may be explained by greater heterogeneity of habitats and environmental conditions (temperature, wave action, productivity, etc.) in shallow areas compared with deeper depths. A strong connection between biotic variability and environmental heterogeneity has also been observed in coral reefs [24] and soft-sediment macrofauna [39], [11], as well as in amphibians, birds and mammals [13].

Overall, the beta diversity of groundfishes decreased with latitude (Fig. 3), although these latitudinal effects diminished sharply with depth. Clarke and Lidgard [42] also observed a negative relationship between the beta diversity of bryozoans and latitude at depths of 10–75 m, but not for shallower or deeper depths up to 200 m. Our analyses based on the probabilistic Raup-Crick measure, taking variation in alpha diversity into account, still yielded a clear pattern of decreasing beta diversity with increasing latitude for shallow depths (<200 m). In terrestrial systems, application of null models indicated that the observed trend of decreasing beta diversity with latitude may be a simple consequence of the species available in the regional pool [37]. Specifically, gamma diversity decreases with latitude at a faster rate than does alpha diversity, which alone may explain the trend in beta diversity. Generally, negative relationships between alpha and/or gamma diversity and latitude have also been reported for marine systems [35], [69], although the strength of this relationship varies with spatial scale and the particular organisms being examined. Meta-analyses by Soininen et al. [14] reported a negative but weak relationship between beta diversity and latitude, which was also weaker in marine systems compared to terrestrial systems. The application of null models to assess the potential for gamma diversity to drive patterns in beta diversity, such as that observed here in groundfish assemblages, or in any marine system, remains to be explored.

### Patterns in beta diversity with depth

The beta diversity of groundfishes, overall, decreased with depth. The deep sea is more environmentally homogeneous than shallow coastal systems in terms of temperature, light, salinity and nutrients, which may explain greater biotic homogeneity being found there as well [44]. Although patterns of richness (alpha or gamma diversity) with depth are highly variable and tend to be context-specific [70], [71], relative abundances of organisms tend to decrease with depth [72], [73], and the richness and evenness of these Pacific groundfish assemblages have been shown to decrease overall with depth [52]. There may be reasonably high biogeographic or historical connectivity of assemblages in the deep sea, including through larval dispersal in deep-sea currents [74]. This contrasts with the suggestion that increased richness in the deep sea is driven by greater localised endemicity [75]. However, increased beta diversity in the deep sea might also be apparent if it were measured at larger (ocean-wide) scales, as historical or contemporary barriers to dispersal among deep basins may contribute to regional endemism [76].

Superimposed on this overall trend of decreasing beta diversity with depth, observed across all latitudes, were two important features: (i) an intermediate peak in beta diversity around 400–600 m depth and (ii) an increase in beta diversity in the deepest zones sampled here, around 1000–1200 m – both of which match previously identified shifts in groundfish assemblage structure in these depth zones [46]. Although, historically, many authors have suggested that broad-scale patterns in biodiversity (primarily alpha and/or gamma diversity) are driven by solar radiation and temperature [5], [6], [31], [52], patterns in beta diversity might have other environmental and historical drivers. For example, a potentially important oceanographic feature of the north-eastern Pacific Ocean is the oxygen minimum zone (OMZ), having oxygen concentrations <0.5 ml·l^−1^. The OMZ occurs at depths of ∼650–1100 m off the coast of California and Oregon [77], [78]. The peaks in biotic heterogeneity around 600 m and 1100 m observed in the present study could well correspond to areas of transition into and out of the OMZ. Dover sole and longspine thornyhead, which are known to be tolerant of low oxygen levels, make up the majority of the catch in the 600–900 m depth zones [52]. In addition, biogenic structures can provide a variety of habitats for groundfishes [79], including potential nursery areas for rockfishes (*Sebastes* spp.) among sponges or corals, in the more oxygen-rich areas bordering the OMZ [80]. The fact that the specific depth at which this intermediate peak in beta diversity occurred decreased with decreasing latitude could be due to a shift in the boundary of the OMZ with changes in latitude [81].

Importantly, the strength of the distance-decay relationship was much stronger for depth than it was for latitude, as evidenced by the difference in the sizes of the Mantel correlations (cf. Table 2 and Table 3). Thus, turnover in assemblage structure is much more intense for the strongly environmentally structured variable of depth, compared to the spatial effects of latitude, even when the latter is considered across shallow depths. Although depth itself is simply a proxy for a host of physical features, including light, pressure and temperature, changes in depth over small distances have important and immediate biological consequences for the physiological tolerances of organisms, compared to the spatial variation in parameters at even very large distances within any given depth [45]. Whereas latitudinal beta diversity is likely to reflect oceanographic transitions and historical factors, turnover with depth is more likely to reflect strong selective environmental filters acting simultaneously. The finding of greater turnover with depth than with latitude at a given spatial scale mirrors findings in terrestrial systems, where elevation is often found to be highly significant in determining ecological turnover [13], [40]. Terrestrial studies suggest turnover along elevational gradients have a strong functional basis and are strongly deterministic by reference to changing environmental conditions and species' tolerances [82], [83]. It is highly likely that the drivers of variation in beta diversity in the ocean along earth's third dimension (i.e., with depth) will also have this strong deterministic (niche) signal of functional significance [84].

### Interactive effects of latitude and depth on beta diversity

Tolimieri and Levin [46] found a clear interaction between depth and latitude with respect to fish assemblage structure. Our results here demonstrate that this interaction is not just in terms of *mean* assemblage structure (i.e., differences among assemblages at different latitudes is greater at shallower depths than at deeper depths), but also *variation* in assemblage structure: namely, the degree of *heterogeneity* in assemblages decreases with depth, *and* this effect is stronger at lower latitudes.

The implications of these results for ecosystem-based fisheries management, as well as for the overall conservation of biodiversity, are many. First, restricting focus to measures of richness or evenness – preserving only so-called “biodiversity hotspots” – does not necessarily ensure adequate management of bio-resources or diversity [85]. Measures of beta diversity can reflect high heterogeneity of habitats, or essential zones of turnover, and preservation of a variety of habitats and niches, including edges and boundaries, has long been an appropriate management goal [17], [32], [86], especially in the absence of more detailed species-specific distributional information.

For groundfishes along the U.S. Pacific coast, we now have evidence that: (i) species richness, density and evenness are lowest in the 600–900-m depth range [52]; (ii) the average taxonomic distinctness (which can reflect greater functional diversity) is highest around 500 m, and (iii) variation in taxonomic distinctness (which can reflect clusters of unrelated but highly-specialised species, as suggested in Zintzen et al. [44]) was highest around 300 m [56]. The current study provides additional highly relevant biodiversity information: namely, that beta diversity, whilst being highest in shallow depths (<200 m), also showed clear peaks at depths around 400–600 m as well as around 1000–1200 m. These peaks were especially marked when differences in area and richness were taken into account (Fig. 5b). Clearly, any plans regarding the development of representative marine reserve networks to enhance either fishery outcomes or conservation benefits would need to integrate all of this information, as well as potential ontogenetic changes in habitat requirements of fishes [87]. For example, areas of high beta diversity documented here indicate areas of high biotic variation, important to protect because they reflect a high diversity of types of assemblages and/or habitats. Furthermore, interactions in these effects of depth with latitude for virtually all of these diversity measures (see also [46]) strongly suggest that both regionally-specific and inter-regional (national-scale) management plans may be an imperative for eventual successful management outcomes.

The fundamental descriptions of patterns in beta diversity we have given here provide an essential framework for the development of appropriate hypotheses regarding the potential forces structuring fish assemblages at global spatial scales. We consider that forward steps for both applied and theoretical advances in our understanding of the ecology of marine systems will include analyses of functional and phylogenetic, as well as taxonomic biodiversity. The use of null models [37], [51] especially in tandem with analyses of temporal variability and functional or genetic traits [88], [89], is likely to yield important insights into the historical, present and future mechanisms governing biodiversity in these marine communities.
